# Machine learning and bioinformatics framework integration reveal potential characteristic genes related to immune cell infiltration in preeclampsia

**DOI:** 10.3389/fphys.2023.1078166

**Published:** 2023-06-13

**Authors:** Lilian Bai, Yanyan Guo, Junxing Gong, Yuchen Li, Hefeng Huang, Yicong Meng, Xinmei Liu

**Affiliations:** ^1^ Shanghai Key Laboratory of Embryo Original Diseases, The International Peace Maternity and Child Health Hospital, School of Medicine, Shanghai Jiao Tong University, Shanghai, China; ^2^ Obstetrics and Gynecology Hospital, Institute of Reproduction and Development, Fudan University, Shanghai, China; ^3^ Research Units of Embryo Original Diseases, Chinese Academy of Medical Sciences, Shanghai, China; ^4^ Key Laboratory of Reproductive Genetics, Ministry of Education, Department of Reproductive Endocrinology, Women’s Hospital, Zhejiang University School of Medicine, Hangzhou, China

**Keywords:** preeclampsia, machine learning, characteristic genes, immune cell infiltration, placental biomarkers

## Abstract

**Introduction:** Preeclampsia is a disease that affects both the mother and child, with serious consequences. Screening the characteristic genes of preeclampsia and studying the placental immune microenvironment are expected to explore specific methods for the treatment of preeclampsia and gain an in-depth understanding of the pathological mechanism of preeclampsia.

**Methods:** We screened for differential genes in preeclampsia by using limma package. Gene Ontology, Kyoto Encyclopedia of Genes and Genomes, disease ontology enrichment, and gene set enrichment analyses were performed. Analysis and identification of preeclampsia biomarkers were performed by using the least absolute shrinkage and selection operator regression model, support vector machine recursive feature elimination, and random forest algorithm. The CIBERSORT algorithm was used to analyze immune cell infiltration. The characteristic genes were verified by RT-qPCR.

**Results:** We identified 73 differential genes, which mainly involved in reproductive structure and system development, hormone transport, etc. KEGG analysis revealed emphasis on cytokine–cytokine receptor interactions and interleukin-17 signaling pathways. Differentially expressed genes were dominantly concentrated in endocrine system diseases and reproductive system diseases. Our findings suggest that *LEP, SASH1, RAB6C*, and *FLT1* can be used as placental markers for preeclampsia and they are associated with various immune cells.

**Conclusion:** The differentially expressed genes in preeclampsia are related to inflammatory response and other pathways. Characteristic genes, *LEP, SASH1, RAB6C*, and *FLT1* can be used as diagnostic and therapeutic targets for preeclampsia, and they are associated with immune cell infiltration. Our findings contribute to the pathophysiological mechanism exploration of preeclampsia. In the future, the sample size needs to be expanded for data analysis and validation, and the immune cells need to be further validated.

## 1 Introduction

Preeclampsia, which is defined as new-onset hypertension and proteinuria after 20 weeks of pregnancy, impaired organ function, or subjective symptoms of preeclampsia in the absence of proteinuria, affects 2%–8% of all pregnancies in developed countries (The American College of Obstetricians and Gynecologists, 2020). Three-fifths of maternal deaths in the United States can be prevented and are often linked to missing diagnoses or delayed diagnostics (Petersen et al., 2019). Preeclampsia is a systemic hypertensive disorder that complicates pregnancy and is caused by placental abnormalities and systemic inflammation. Preeclampsia can lead to maternal, fetal, and infant mortality (Hutcheon et al., 2011; Lisonkova and Joseph, 2013). Studies have shown that there is a genetic predisposition for preeclampsia and that it is significantly associated with genetic variants associated with thrombosis, infection, oxidative stress, and the renin-angiotensin system (Williams and Broughton Pipkin, 2011; Jebbink et al., 2012; Rana et al., 2014). Therefore, screening genes characteristic of preeclampsia is expected to help explore specific treatment methods, gain insight into the pathological mechanisms of preeclampsia, and contribute to the use of tools to help determine pregnant women who are at high risk of preeclampsia before clinical manifestations (McCarthy et al., 2018; Henderson et al., 2021).

Recently, bioinformatics analysis has been used to identify new genes as biomarkers for disease diagnosis and prognosis (Cao et al., 2019). “Machine learning” generally refers to the process of fitting prediction models to data or identifying information grouping in data. Machine learning is essentially an attempt to mimic the ability of humans to recognize patterns through computation but in a more objective way (Greener et al., 2022). For preeclampsia, early detection improves prognosis, but there are currently no reliable screening tests to predict its development, especially in term pregnancy when the disease burden is greatest. Many potential biomarkers have been identified through exploratory studies using established disease samples. Combining biomarkers from multiple organ and cellular sources may yield the best predictive results (MacDonald et al., 2022). To this end, researchers have been exploring first-trimester biochemical markers that may help identify women at risk of developing hypertensive disorders of pregnancy. One study found that the placental immune function of patients with preeclampsia was altered. Proteasomes, spliceosomes, ribosomes, and mitochondria were abnormally active in the new villi cytotrophoblast cell types (Zhang et al., 2021). In addition, a protein encoded by a differentially expressed mRNA in maternal serum, Follistatinlike 3 (FSTL3), has been reported to be able to predict preeclampsia and FGR (Gong et al., 2021). Notably, studies have been conducted to measure circulating cell-free RNA (cfRNA) by liquid biopsy to study the development of pregnancy-related complications in a non-invasive manner, and they have demonstrated that cfRNA measurement can predict preeclampsia in early pregnancy (Moufarrej et al., 2022). Similarly, another study showed that cfRNA signatures from a single blood draw can track pregnancy progression at the placental, maternal, and fetal levels and robustly predict preeclampsia (Rasmussen et al., 2022). Despite significant progress in early prediction of preeclampsia risk, elucidation of the pathogenesis of preeclampsia remains a critical and ongoing area of research. The uncovering of pathogenesis will help to better understand the development of preeclampsia and allow for better diagnosis and treatment of the disease.

Recent research has suggested that immune cell infiltration plays an important role in the development of preeclampsia (Aneman et al., 2020). The maternal–fetal interface is composed of decidual stromal, decidual immune, and trophoblast cells (Yang et al., 2019). In early pregnancy, precise regulation of the maternal immune system aids in the successful implantation of the placenta (LaMarca et al., 2013). Maintenance of a normal pregnancy requires a balanced state of immune cells and cytokines from the maternal-fetal interface, and an unbalanced immune response can result in abnormal placental structure or angiogenesis (Sahay et al., 2014; LaMarca et al., 2016). Fortunately, emerging technologies have the potential to indicate imbalances that may lead to conditions such as preeclampsia. CIBERSORT is a method that quantifies the proportion of immune cells in preeclamptic and normal tissue samples based on gene expression profiles (Newman et al., 2015).

**FIGURE 1:**
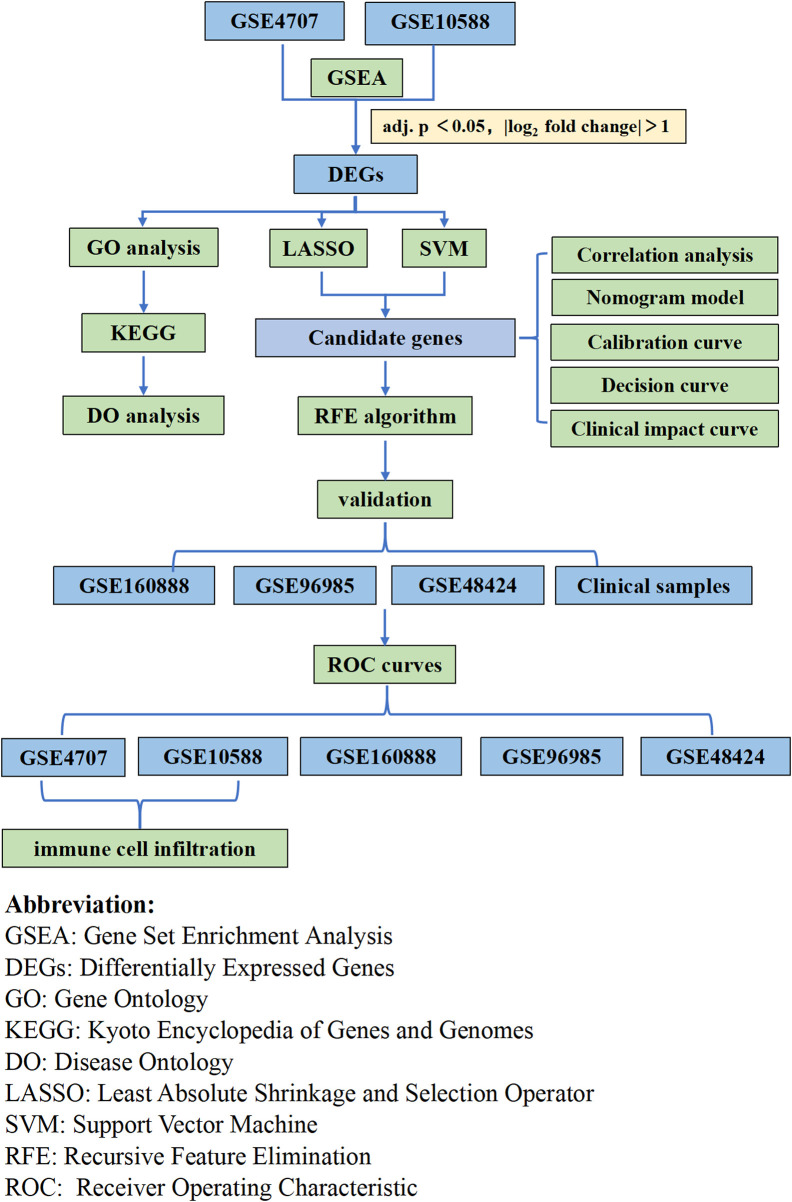
Flow chart of the whole analysis.

In the present study, we explored the relationship between immune cell infiltration and preeclampsia using CIBERSORT and the differences in immune cell infiltration between preeclamptic and normal pregnant women. We utilized two preeclampsia microarray datasets from the Gene Expression Omnibus (GEO) database and analyzed the differentially expressed genes (DEGs). We used a machine learning algorithm to screen and determine placentabiomarkers, and then validated these immune infiltration-related candidate genes in other cohorts. Then, we used placental samples from pregnant women to verify the predictions. Our analysis approach is shown in Figure 1. Herein, we discuss the association between the identified biomarkers and infiltrating immune cells. Our findings provide new insights for the exploration of the mechanisms underlying the development of preeclampsia and new insights for its diagnosis and treatment.

## 2 Materials and methods

### 2.1 Microarray data

The GSE4707 and GSE10588 datasets were downloaded from the GEO database (http://www.ncbi.nlm.nih.gov/geo/). GSE4707 was based on GPL1708, with an Agilent-012391 Whole Human Genome Oligo Microarray G4112A (Feature Number version), and placental biopsies were obtained from ten patients with preeclampsia and four women with normal pregnancies during Caesarean section. Chorionic tissue was dissected from a standardized location approximately 2 cm beside the umbilical cord insertion, from the middle layer of the placenta midway between maternal and fetal surfaces. In the GSE4707 dataset, there was no statistical difference in gestational age and weight in patients with preeclampsia compared with normal mothers, but neonatal weight in the preeclampsia group was significantly lower than neonatal weight in the normal group. GSE10588 was based on GPL2986 using ABI Human Genome Survey Microarray Version 2, and placental biopsies were obtained from 17 patients with preeclampsia and 26 women with normal pregnancies. A central area of chorionic tissue was dissected, and the maternal deciduas and amnionic membranes were removed. Scientists then dissected 1-cm-thick sections of placental villi from the central area between basal and chorionic plates. In the GSE10588 dataset, there were no statistical differences in maternal age, BMI, but patients with preeclampsia had lower gestational age and higher cesarean delivery rates compared to normal mothers. The probe in each data file was modified to a gene symbol according to its annotations in the probe file. If the same gene symbol corresponded to various probes, the diameter of the probe was assessed as the final expression value. Then, the results were merged for further analysis. Simultaneously, the function of the “SVA” package of R version 4.2.0 was used to eliminate the batch effect (Leek et al., 2012). We used the GSE160888 dataset according to Agilent-045997 Arraystar human lncRNA microarray V3, which included placental samples from four preeclampsia and four control cases. The placenta specimen was resected from the middle of the villous lobule. We also used GSE96985 based on Agilent-078298 human ceRNA array V1.0 4 × 180K, which included placental samples from four preeclampsia and three control cases. Using the method mentioned above, both datasets were combined as a validation cohort. The GSE48424 dataset, which was based on Agilent-014850 Whole Human Genome Microarray 4 × 44K G4112F and includes whole blood samples from 18 preeclampsia and 18 control cases, was also downloaded.

### 2.2 Data processing and DEG filtering

The two datasets were combined into a metadata queue, and the SVA package was used to eliminate the batch effect of the two datasets. The R limma package (http://www.bioconductor.org/) was used for background correction, normalization, and differential expression between array analyses. We used a false discovery rate-adjusted sample (false discovery rate adjusted; *p* < 0.05) and | log2 Fold Change | > 1 as the threshold point for DEGs.

### 2.3 Functional enrichment analysis

To determine the main biological properties of DEGs, Gene Ontology (GO) and Kyoto Encyclopedia of Genes and Genomes (KEGG) enrichment analyses were performed. The R packages “ggplot2” (Wickham, 2011), “enrich Plot”, and “clusterProfiler” (Yu et al., 2012) were used to create GO and KEGG enrichment plots. Disease ontology (DO) enrichment analysis was also conducted on DEGs using “clusterProfiler” and the “DOSE” software package in R (Yu et al., 2012; Yu et al., 2015). Through Gene Set Enrichment Analysis (GSEA), the most notable functional differences between the preeclampsia and control groups were verified (Subramanian et al., 2005). The study set c2.cp.go.v7.0. symbols, and the GMT from the Molecular Marker Database (MSigDB) were used as reference gene sets (Liberzon et al., 2015).

### 2.4 Diagnostic screening and correlation analysis of candidate biomarkers

We used three machine-learning algorithms to screen for important biomarkers of preeclampsia. The least absolute shrinkage and selection operator (LASSO) is a regression analysis algorithm that uses regularization to increase prediction accuracy. In the “glmnet” software package in R, the LASSO regression algorithm was used to select preeclampsia genes that were notably related to normal samples (Engebretsen and Bohlin, 2019). Support vector machine (SVM) is a popular machine-learning technique for classification and regression (Nedaie and Najafi, 2018). We used “E1071,” “Kernlab,” and “caret” to build the SVM model. To avoid overfitting, we determined the optimal genes from the metadata queue using the Recursive feature elimination (RFE) algorithm. The LASSO and SVM were used to select overlapping genes, and the level of candidate genes was verified on the GSE160888 and GSE96985 datasets. In addition, we established a random forest (RF) model in the “RandomForest” package in R as a training model to forecast the occurrence of preeclampsia. Moreover, ntrees and mtry were set at 100 and 3, respectively. Next, a rosette model was built with the “RMS” package in R to forecast the prevalence of patients with preeclampsia. Calibration curves were used to determine the agreement between predicted and actual values. A clinical influence curve was drawn using decision curve analysis (DCA) to evaluate whether model-based decision-making is beneficial to patients (Iasonos et al., 2008). We used the “limma”, “ggplot2”, “ggpubr”, and “ggExtra” packages in R to calculate and plot the correlations between the four characteristic genes.

### 2.5 Diagnostic value of characteristic biomarkers in preeclampsia

To validate the predictive value of previously screened biomarkers, receiver operating characteristic (ROC) curves were built via the “pROC” package based on mRNA expression data using 30 preeclampsia and 27 control samples. The area under the ROC curve (AUC) was used to judge the diagnostic efficiency of preeclampsia and control samples, which was further verified using the GSE160888, GSE96985, and GSE48424 data files.

### 2.6 Discovery of immune cell subtypes

The CIBERSORT bioinformatics (https://cibersortx.stanford.edu/) algorithm was used to analyze immune cell infiltration from the preeclampsia gene expression profiles. Putative immune cell abundance was predicted using a reference set of 22 immune cell subtypes with 1000 permutations (LM22; Newman et al., 2015). Correlation analysis and visualization of 21 infiltrating immune cells were performed using the R package “Corrplot.” The Vioplot software package was used to draw a violin diagram to observe the difference in immune cell infiltration between patients with preeclampsia and normal pregnant women.

### 2.7 Analysis of correlation between identified genes and infiltrating immune cells

Pearson’s correlation analysis was performed using R to determine the correlation between the identified gene biomarkers and the level of infiltrating immune cells. The resulting associations were visualized using the graphical techniques of the “ggplot2” package.

### 2.8 Patient enrollment and data collection

Twenty-one pregnant women were selected from the International Peace Maternal and Child Health Hospital affiliated to Shanghai Jiao Tong University School of Medicine. Nine cases were diagnosed as preeclampsia and twelve cases were normal pregnant women. Preeclampsia was diagnosed according to the ACOG Practice Bulletin (The American College of Obstetricians and Gynecologists, 2020). All participants were singleton pregnant women without other diseases affecting blood pressure, such as hyperthyroidism, Cushing’s syndrome, and pancreatitis; serious dysfunction of the heart, liver, and kidney; and acute complications, such as diabetic ketoacidosis. All pregnant women provided signed informed consent, and this study was approved by the Ethics Committee of International Peace Maternal and Child Health Hospital Affiliated to Shanghai Jiao Tong University School of Medicine [Approval No.: GKLW-2017-81].

General information on the pregnant women, including age at delivery and family history of hypertension, was collected through face-to-face interviews. The pre-pregnancy body-mass index was calculated from the height measured by nurses and the pre-pregnancy weight reported by the pregnant women. Blood pressure was measured during the second trimester.

The amniotic membrane was removed within 5 min after delivery of the placenta. We dissected the middle layer of the placenta from the middle of the maternal and fetal surfaces, followed by washing with enzyme-free water to remove blood. Then, the water was removed using filter paper, and the placenta was quickly placed in an external rotating freezing tube, flash-frozen in liquid nitrogen, and stored at −80°C for later use.

### 2.9 Expression of four characteristic genes in placenta of control and preeclampsia groups

The total RNA of the placenta was extracted using RNAiso Plus reagent (9109, Takara, Shiga, Japan) according to the manufacturer’s instructions. cDNA was synthesized from RNA using the RT Reagent Kit and gDNA Eraser (RR047A, Takara, Shiga, Japan). qPCR was performed on the QuantStudio 7 Flex system (Life Technologies, Carlsbad, CA, United States). Three replicates of each sample were analyzed. To quantify the relative mRNA expression, data were normalized to the expression level of β-actin. Primer sequences are shown in Table 1.

**TABLE 1:** Sequences of primer sets for qRT-PCR.

Gene	Forward primer	Reverse primer
Leptin	TTC​TTG​TGG​CTT​TGG​CCC​TA	TGG​ATA​AGG​TCA​GGA​TGG​GGT
FLT1	ATT​CCG​AAG​CAA​GGT​GTG​ACT	AGA​AGC​TTG​TAG​GTG​GCA​ACA
SASH1	GGC​CGG​AAG​TTG​GTC​AAA​AC	CAG​GTT​CTC​CCG​TGG​CTT​AG
RAB6C	TGA​AGA​CGG​AAG​ACG​GAA​GAC	CCA​AAA​CCA​GCC​TGA​AAG​ACC
ACTIN	GTC​CAC​CGC​AAA​TGC​TTC​TA	TGC​TGT​CAC​CTT​CAC​CGT​TC

### 2.10 Statistical analysis

R and SPSS26.0 were used for statistical analyses. Student’s t-test and Mann-Whitney test were used for continuous, normally and non-normally distributed data, respectively. The “glmnet” and E1071 packages in R and ROC curve analysis were used for the LASSO regression algorithm and the diagnostic efficacy of the selected biomarker analysis, respectively. Pearson’s correlation coefficient was used to study the relationship between gene biomarker expression and immune cell infiltration. For quantitative data, distribution was described in terms of mean ± standard deviation, and an independent sample t-test was used for comparison between groups. The median M [P25, P75] was used to describe data that did not conform to normal distribution, and the rank sum test of independent samples was used for comparison between groups. Enumeration data were described in terms of the number of cases (%), and the Fisher’s exact probability method was used for comparison of differences between groups. All statistical analyses were bilateral, and *p* < 0.05 was considered to indicate significance.

## 3 Results

### 3.1 Identification of DEGs in preeclampsia

**FIGURE 2:**
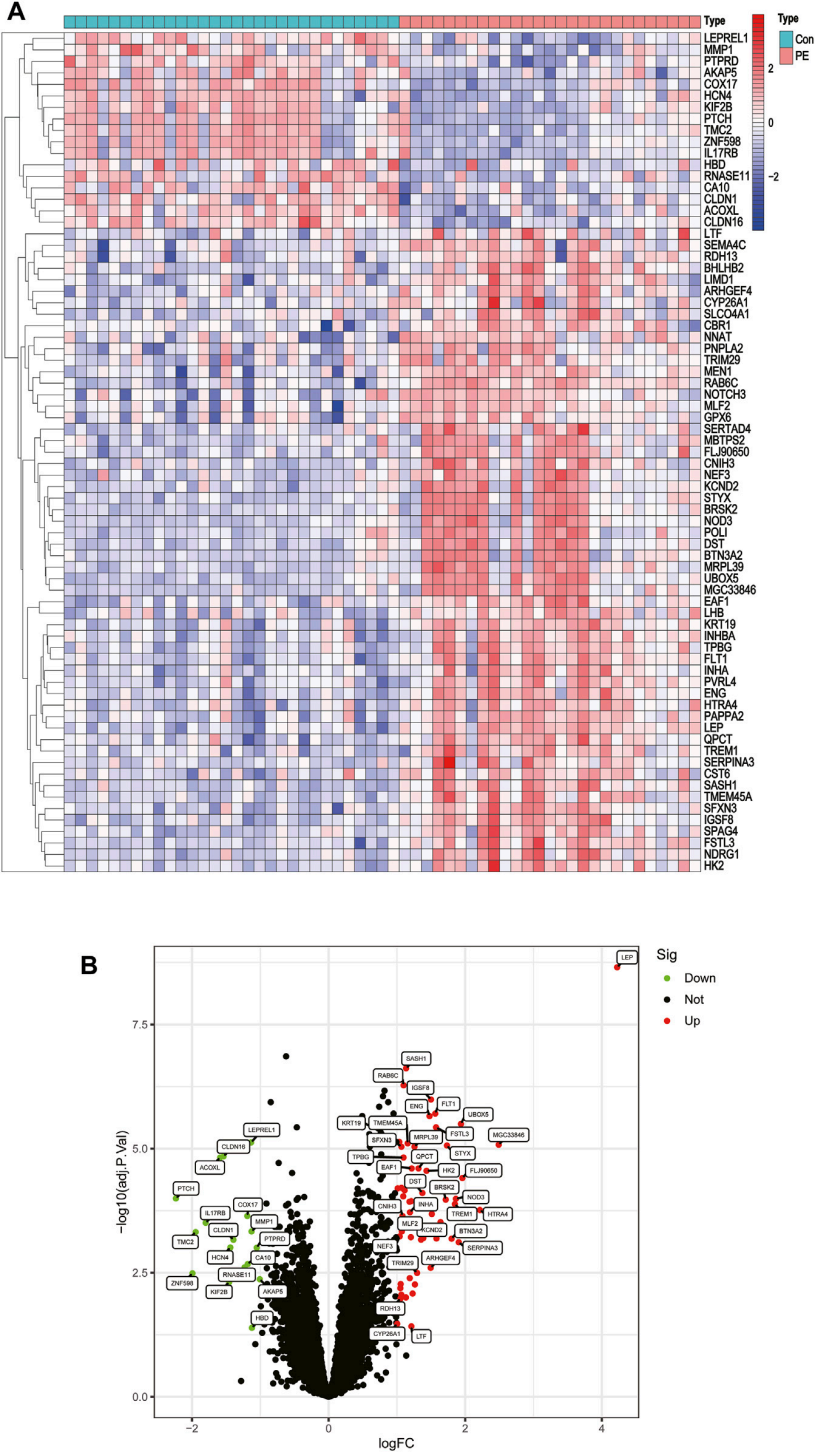
Differentially expressed genes between placentas of patients with preeclampsia and controls. **(A)** Heatmap. **(B)** Volcano plot.

Two GEO datasets (GSE4707 and GSE10588) were downloaded, which together included 27 patients with preeclampsia and 30 normal pregnant women. After eliminating batch sub-effects, the “limma” package was used to determine the DEGs. We created heat maps with the screened differential genes, with red representing upregulated genes in PE patients and blue representing downregulated genes (Figure 2A). We also plotted volcano plots, with red representing upregulated genes in PCOS patients and green representing downregulated genes (Figure 2B). Seventy-three DEGs were identified, of which 56 were significantly upregulated and 17 significantly downregulated.

### 3.2 Functional correlation analysis

**FIGURE 3:**
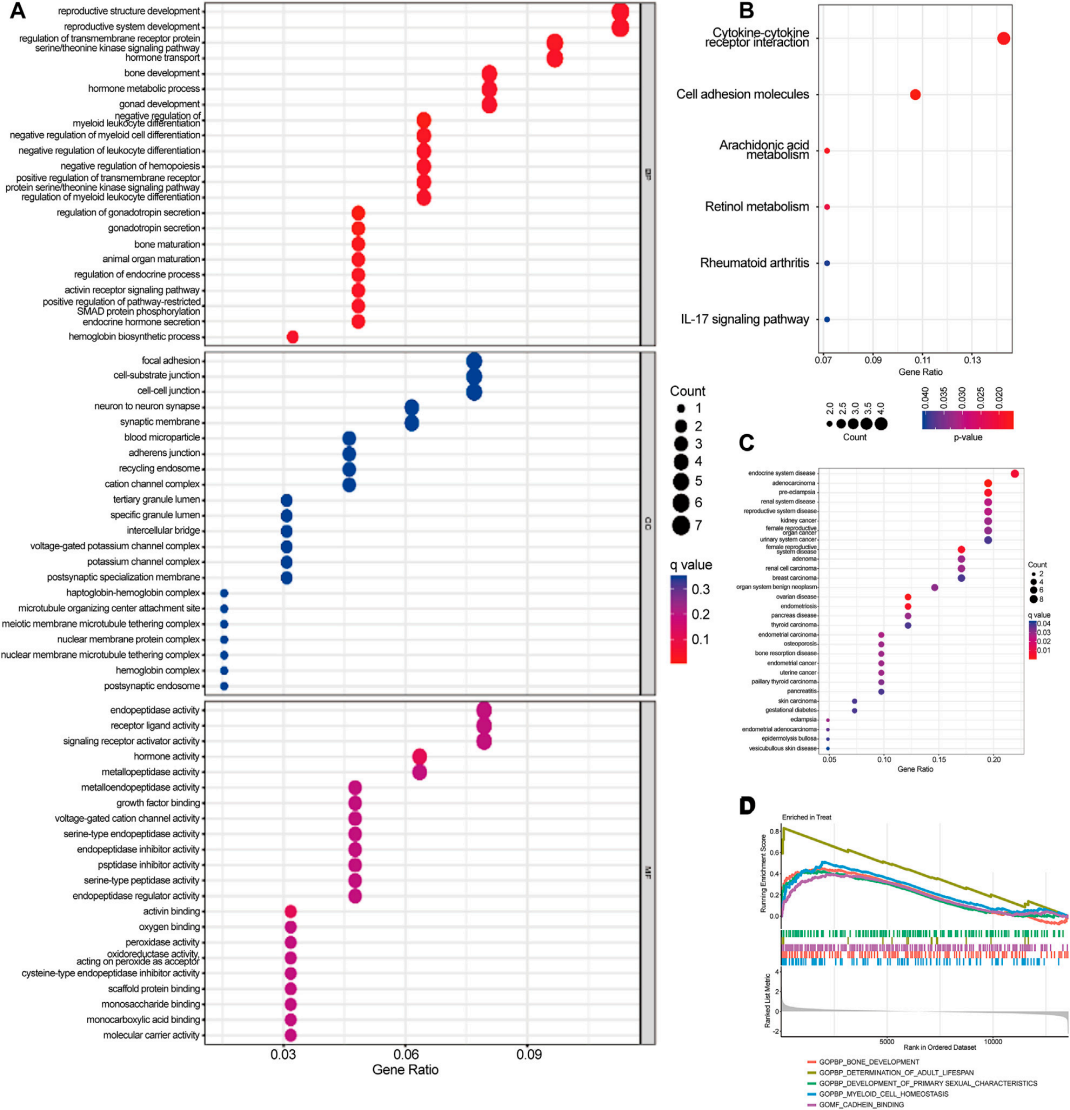
Gene Ontology (GO), Kyoto Encyclopedia of Genes and Genomes (KEGG), Disease Ontology (DO), and Gene Set Enrichment Analysis (GSEA) were used to identify the underlying biological processes of differential genes. **(A)** GO analysis of differential genes between preeclampsia and control samples. **(B)** KEGG analysis of differential genes between preeclampsia and control samples. **(C)** DO enrichment analysis of differentially expressed genes between preeclampsia and control samples. **(D)** Enrichment analysis by GSEA.

GO analyses showed that DEGs were predominantly enriched in reproductive structure development, reproductive system development, and hormone metabolic processes (Figure 3A). KEGG analyses indicated that DEGs were predominantly enriched in cytokine-cytokine receptor interaction, cell adhesion molecules, and the interleukin (IL)-17 signaling pathway (Figure 3B). DO pathway enrichment analyses suggested that diseases enriched by DEGs were largely associated with endocrine system diseases, preeclampsia, and reproductive system diseases (Figure 3C). GSEA results of preeclampsia showed that the enriched pathways dominated myeloid cell homeostasis and cadherin binding (Figure 3D).

### 3.3 Identification and validation of biomarkers of diagnostic properties

**FIGURE 4:**
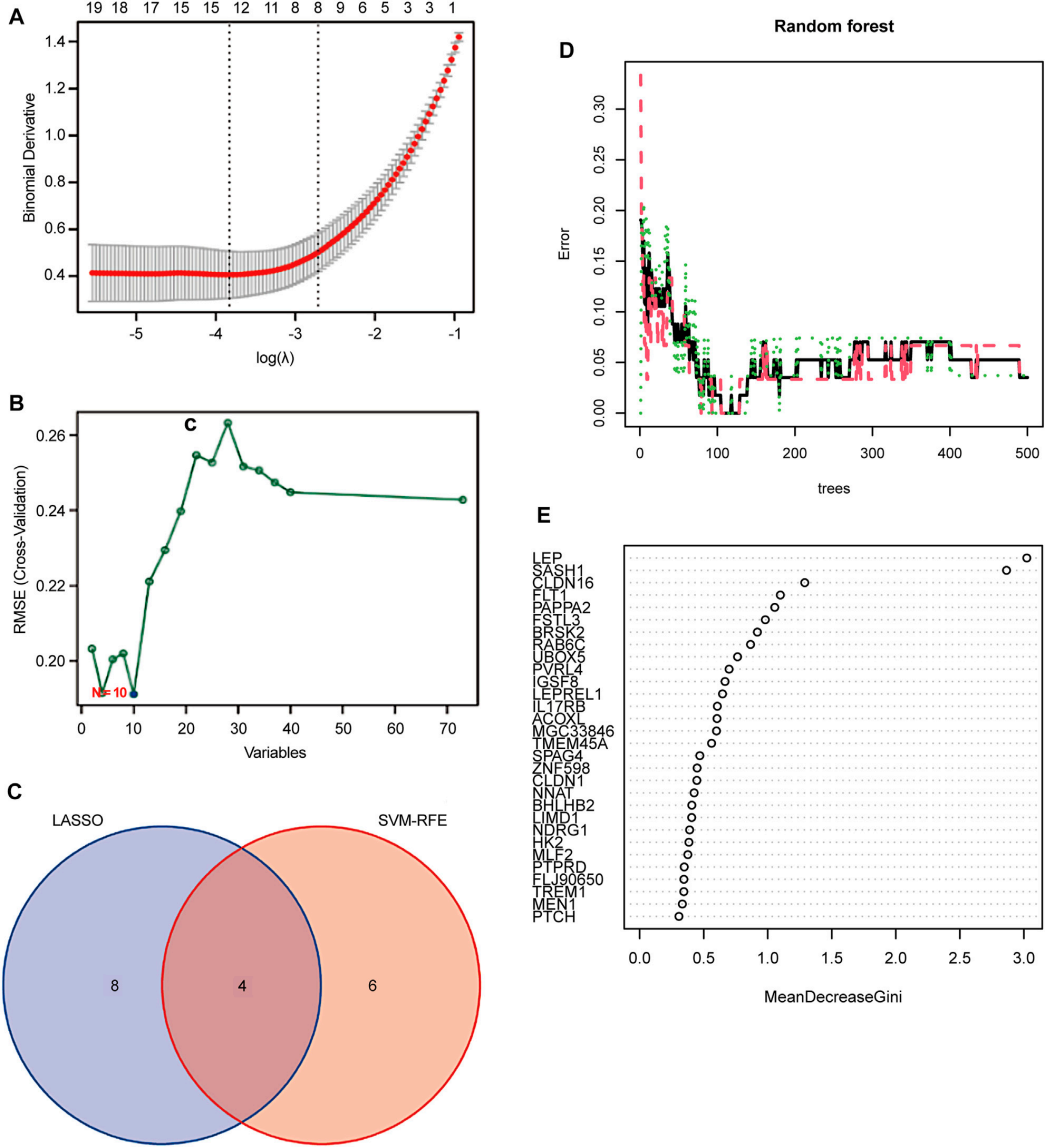
Screening candidate biomarkers for the diagnosis of preeclampsia. **(A)** Adjustment of feature selection in the least absolute shrinkage and selection operator model (LASSO). **(B)** Plot of biomarker selection by support vector machine-recursive feature elimination (SVM-RFE) algorithm. **(C)** Venn diagram screening LASSO for four diagnostic genes common to the SVM-RFE algorithm. Random Forest (RF) model construction. **(D)** Inverse cumulative distribution of residuals is plotted to show the residual distribution of RF. **(E)** Importance scores of variables based on the RF model.

**FIGURE 5:**
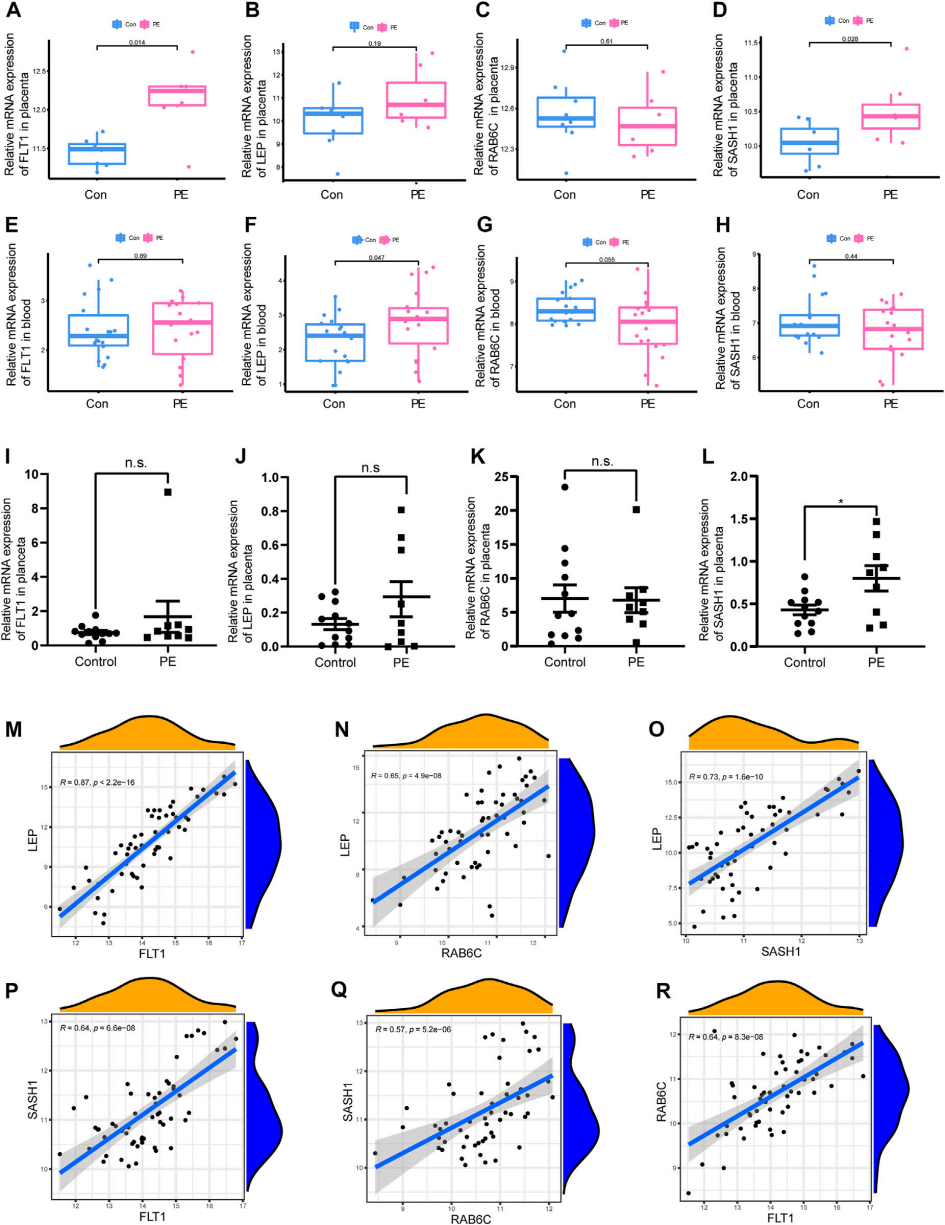
Box plot validation of four candidate diagnostic genes and correlation analysis of four characteristic genes. **(A–D)** Validation of four candidate genes for preeclampsia in human placenta using GSE160888 and GSE96985 datasets. **(E–H)** Validation of four candidate genes in peripheral blood using GSE48424. **(I–L)** RT-qPCR was used to verify the expression of four characteristic genes in the placenta of the patients. **(M–R)** Correlation analysis of the four characteristic genes in GSE4707 and GSE10588.

Two different algorithms were used to identify promising biomarkers of preeclampsia. First, 12 genes were identified as diagnostic biomarkers of preeclampsia using the LASSO algorithm based on the DEGs (Figure 4A). Then, the SVM-recursive feature elimination algorithm was used to screen ten characteristic genes from the DEGs (Figure 4B). Four overlapping features (*leptin* [*LEP*], *SAM* and *SH3 domain containing 1* [*SASH1*], *RAB6C*, and *fms-like tyrosine kinase receptor-1* [*FLT1*]) were selected using the two algorithms (Figure 4C). We also constructed an RF tree and screened out two genes with a score greater than two according to their importance score, namely *LEP* and *SASH1* (Figures 4D, E). We then used the GSE160888 and GSE96985 datasets to analyze their levels. *FLT1* levels in the placenta of patients with preeclampsia were significantly upregulated (*p* = 0.014; Figure 5A). Furthermore, the expression of *LEP* in the tissues of patients with preeclampsia was higher than that in the control group and that of *RAB6C* was lower, but no significant differences were observed (Figures 5B, C). Furthermore, the *SASH1* levels in the placenta of patients with preeclampsia were significantly upregulated (*p* = 0.028; Figure 5D). We then used the GSE48424 dataset, which contained 18 blood samples from patients with preeclampsia and 18 blood samples from control patients, to analyze the expression of these four genes. *FLT1* expression in the blood samples of patients with preeclampsia was higher than that in control patients, but the difference was not significant (Figure 5E). The expression of *LEP* in the blood samples of patients with preeclampsia was significantly higher than that in control patients (*p* = 0.047; Figure 5F); the expression of *RAB6C* was lower in the blood of patients with preeclampsia (Figure 5G). This was consistent with the results validated in the placenta. However, *SASH1* showed the opposite results in the placenta and blood. The expression of *SASH1* was significantly increased in the placenta of patients with preeclampsia but decreased in the blood (Figures 5D, H).

### 3.4 qPCR to verify the expression of four characteristic genes in the placenta of patients

We examined the expression of four genes in nine patients with preeclampsia and 12 normal pregnant women. Basic information on the patients is shown in Table 2. The expression of *SASH1* was significantly increased in patients with preeclampsia both in the predicted and qPCR experiment results (Figures 5D, L). However, we predicted that *FLT1* expression would be significantly increased in preeclampsia (Figure 5A), but there was no significant difference in the qPCR results (Figure 5I). Both the predicted results and the experimental verification showed that the expression of *LEP* in patients with preeclampsia was higher and that of *RAB6C* was lower than those in normal pregnant women, but there was no significant difference (Figures 5B, J).

**TABLE 2:** Demographic data of the study population.

	Control	Preeclampsia	*P*
	***N = 12***	***N = 9***	
Age (years)	30.33 ± 2.50	32.00 ± 4.03	0.295
Pre-pregnancy BMI	20.55 [19.32; 21.02]	23.20 [21.30; 24.50]	0.017
Family history of hypertension			1.000
No	10 (83.33%)	7 (77.78%)	
Yes	2 (16.67%)	2 (22.22%)	
Delivery gestational age (weeks)	38.58 ± 0.90	37.44 ± 1.33	0.045
SBP (mmHg)	112.58 ± 7.62	141.44 ± 8.26	<0.001
DBP (mmHg)	72.50 ± 6.79	84.44 ± 11.48	0.017
Quantification of urinary protein (g/24 h)	-	0.42 [0.20; 1.00]	-
Serum creatinine (μmol/L)	48.32 [43.50; 53.50]	55.00 [46.60; 60.80]	0.286
Aspartate aminotransferase (U/L)	16.50 [13.00; 21.00]	34.00 [18.00; 39.00]	0.009
Alanine aminotransferase (U/L)	8.00 [6.50; 10.00]	24.00 [16.00; 35.00]	0.003
The total protein (g/L)	64.62 ± 3.09	59.68 ± 3.17	0.002
Albumin (g/L)	37.24 ± 3.25	33.50 ± 3.64	0.026
Globulin (g/L)	27.38 ± 3.27	26.18 ± 3.44	0.431

BMI, body mass index; SBP, systolic blood pressure; DBP, diastolic blood pressure.

Values in square brackets are upper and lower quartiles.

### 3.5 Correlation analysis between four characteristic genes

We analyzed the correlations among the expression of *FLT1*, *LEP*, *SASH1*, and *RAB6C* in GSE4707 and GSE10588. *LEP* was positively correlated with *FLT1* (R = 0.87, *p* < 2.2E-16; Figure 5M), *RAB6C* (R = 0.65, *p* = 4.9E-08; Figure 5N), and *SASH1* (R = 0.73, *p* = 1.6E-10; Figure 5O). Furthermore, *SASH1* was positively correlated with *FLT1* (R = 0.64, *p* = 6.6E-08; Figure 5P) and *RAB6C* (R = 0.57, *p* = 5.2E-06; Figure 5Q). Finally, *RAB6C* was positively correlated with *FLT1* (R = 0.64, *p* = 8.3e-08; Figure 5R).

### 3.6Construction of nomogram model

**FIGURE 6:**
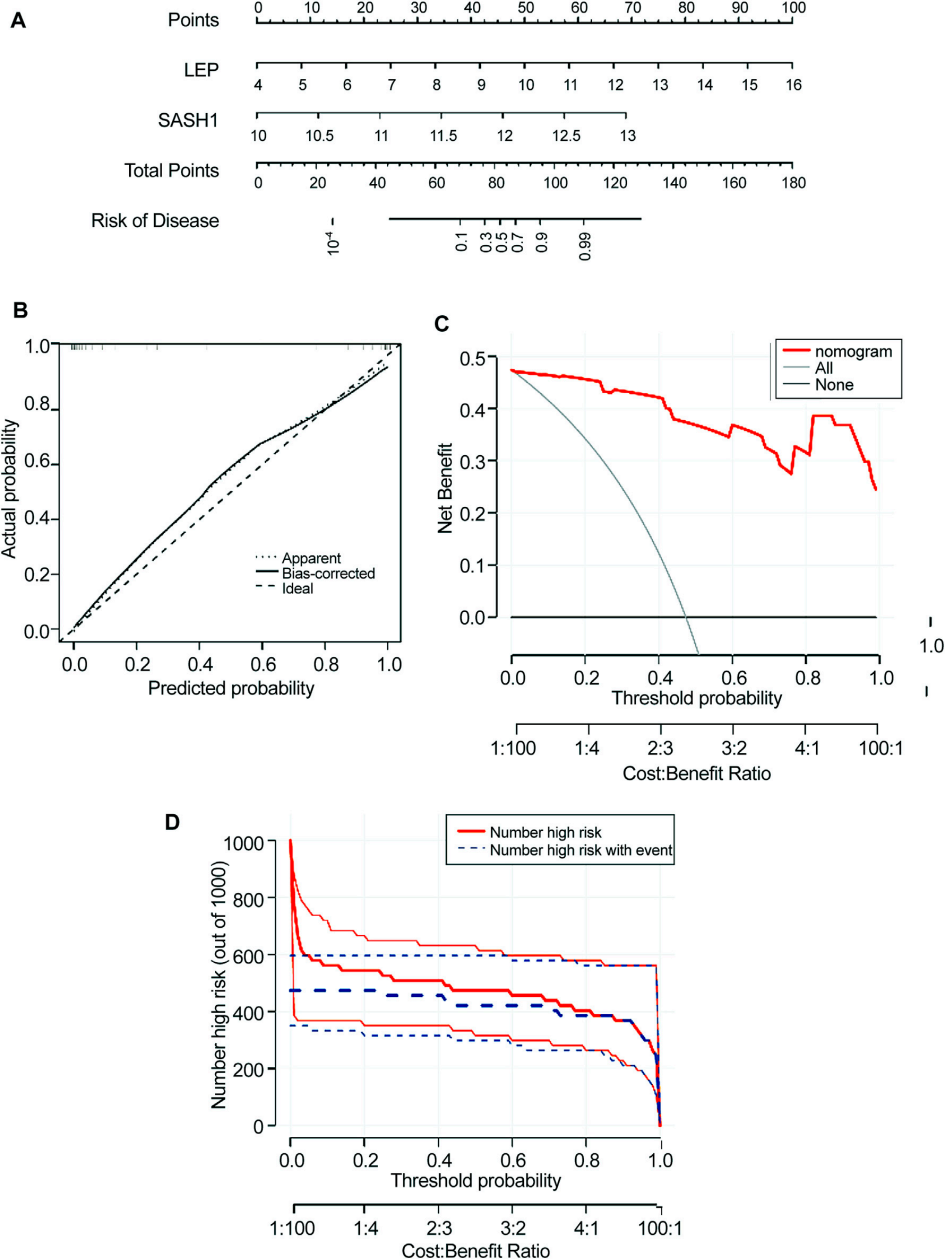
Construction of the nomogram model. **(A)** Construction of a nomogram model based on four candidate genes. **(B)** Calibration curve showing the predictive power of the nomogram model. **(C)** Decision curve. **(D)** Clinical impact of the nomogram model was assessed using the clinical impact curve.

A nomogram model for predicting the prevalence of preeclampsia based on the two candidate genes was constructed using the ‘RMS’ package (Figure 6A). The calibration curve showed that the prediction ability of the nomogram model was optimized (Figure 6B). The red line in the DCA curve remained above the gray and black lines between 0 and 1, indicating that decisions based on the nomogram model may be beneficial to patients with preeclampsia (Figure 6C). The clinical influence curve showed that the nomogram model had a good predictive ability (Figure 6D).

### 3.7 Diagnostic efficacy of functional biomarkers in preeclampsia

**FIGURE 7:**
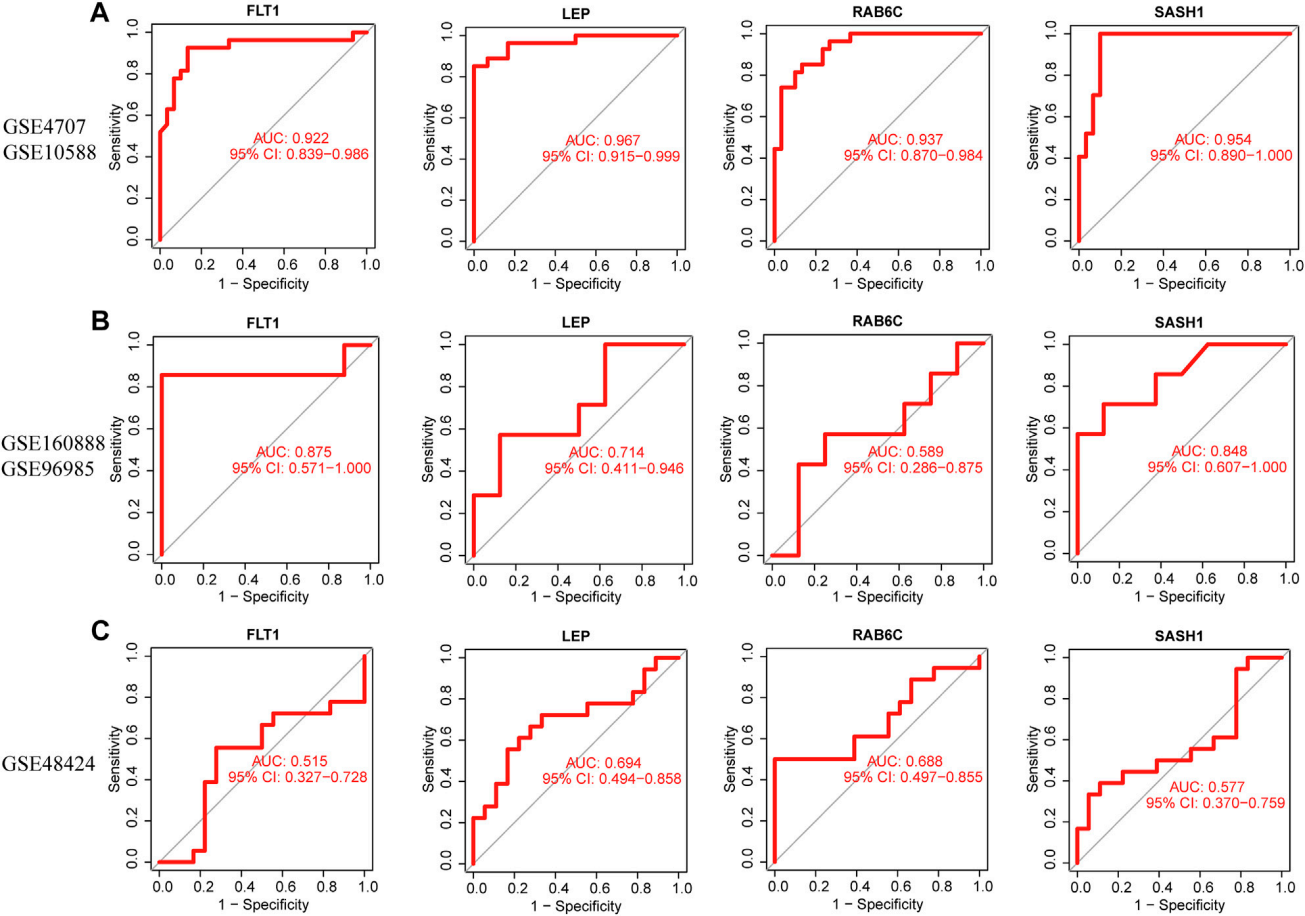
Receiver operating characteristic (ROC) curves of the diagnostic effectiveness of four diagnostic markers. **(A)** *FLT1, LEP, RAB6C*, and *SASH1* fitting metadata cohort of GSE4707 and GSE10588. **(B)** Fitting ROC curves of *FLT1, LEP, RAB6C*, and *SASH1* in the combined dataset of GSE160888 and GSE96985. **(C)** Fitting the ROC curves of *FLT1, LEP, RAB6C*, and *SASH1* in the GSE48424 dataset.

The four biomarkers showed good diagnostic value for preeclampsia and control samples in the GSE4707 and GSE10588 datasets (Figure 7A). The AUC of *FLT1* was 0.922 (95% confidence interval [CI] 0.839–0.986) and that of *LEP* was 0.967 (95% CI 0.915–0.999). The AUC of *SASH1* was 0.954 (95% CI 0.890–1.000) and that of *RAB6C* was 0.937 (95% CI 0.870–0.984). The GSE160888 and GSE96985 datasets verified its recognition ability, with an AUC of 0.875 (95% CI 0.571–1.000) for *FLT1* and 0.714 (95% CI 0.411–0.946) for *LEP*. The AUC of *RAB6C* was 0.589 (95% CI 0.286–0.875) and that of *SASH1* was 0.848 (95% CI 0.607–1.000), indicating that the characteristic biomarkers had diagnostic ability (Figure 7B). The GSE48424 dataset showed verified recognition ability, with an AUC of 0.515 (95% CI 0.327–0.728) for *FLT1* and 0.694 (95% CI 0.494–0.858) for *LEP*. The AUC of *RAB6C* was 0.688 (95% CI 0.497–0.855), and that of *SASH1* was 0.577 (95% CI 0.370–0.759), indicating that the characteristic biomarkers had diagnostic ability (Figure 7C).

### 3.8 Immune cell infiltration

**FIGURE 8:**
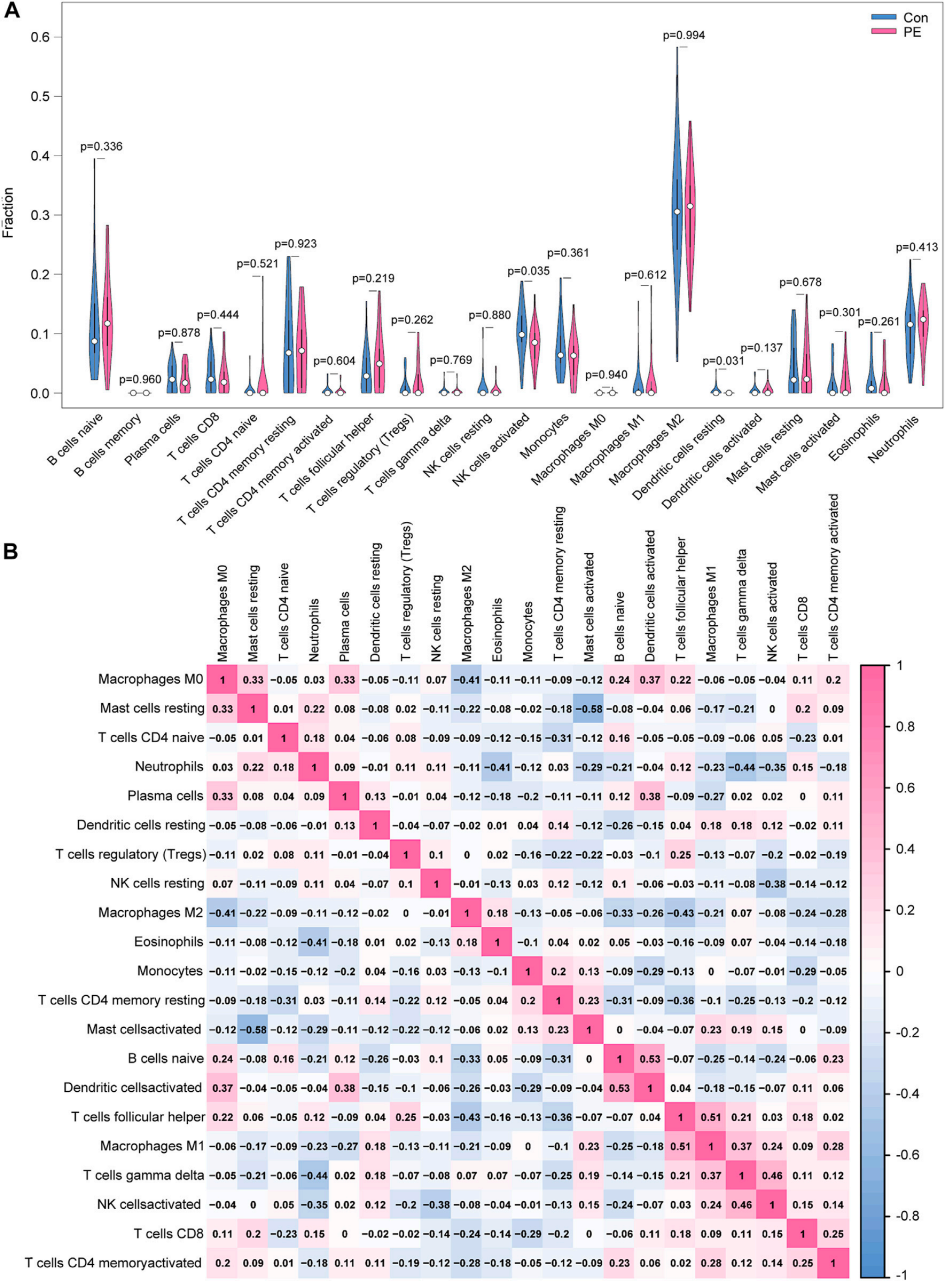
Distribution and visualization of immune cell infiltration. **(A)** Comparison of 22 immune cell subtypes in patients with preeclampsia and controls. Blue and red represent normal and preeclampsia samples, respectively. **(B)** Correlation matrix of 21 immune cell subtypes, with immune cell subtypes shown on the horizontal and vertical axes. Immune cell subtype composition (higher, lower, and same relative levels are shown in red, blue, and white, respectively).

Only activated NK cells and resting dendritic cells were statistically significant (*p* < 0.05; Figure 8A), whereas the other cell types did not differ significantly between the placenta of normal pregnant women and patients with preeclampsia. The correlation between infiltrated immune cells is displayed in Figure 8B; Table 3.

**TABLE 3:** Correlation between immune cells.

Immune cell types	Immune cell types	Correlation coefficient (r)	P
naive B cells	activated dendritic cells	0.53	*p* < 0.001
	M2 macrophages	−0.33	*p* = 0.01
	resting CD4 memory T cells	−0.31	*p* = 0.02
	resting dendritic cells	−0.26	*p* = 0.047
Activated dendritic cells	plasma cells	0.38	*p* = 0.004
	M0 macrophages	0.37	*p* = 0.004
	monocytes	−0.29	*p* = 0.029
Eosinophils	neutrophils	−0.41	*p* = 0.002
M0 macrophages	activated dendritic cells	0.37	*p* = 0.004
	plasma cells	0.32	*p* = 0.01
	resting mast cells	0.33	*p* = 0.01
	M2 macrophages	−0.41	*p* = 0.002
M1 macrophages	gamma delta T cells	0.37	*p* = 0.005
	activated CD4 memory T cells	0.28	*p* = 0.037
	follicular helper T cells	0.51	*p* < 0.001
	plasma cells	−0.27	*p* = 0.04
M2 macrophages	follicular helper T cells	−0.43	*p* < 0.001
	activated CD4 memory T cells	−0.28	*p* = 0.037
Activated mast cells	resting mast cells	−0.58	*p* < 0.001
	neutrophils	−0.29	*p* = 0.028
Neutrophils	gamma delta T cells	−0.44	*p* < 0.001
	activated NK cells	−0.35	*p* = 0.007
	gamma delta T cells	−0.54	*p* < 0.0001
Activated NK cells	gamma delta T cells	0.46	*p* = 0.003
	resting NK cells	−0.38	*p* = 0.004
Resting memory CD4 T cells	follicular helper T cells	−0.36	*p* = 0.007
	naive CD4 T cells	−0.31	*p* = 0.02

### 3.9 Correlation analysis of expression of four biomarkers with abundance of infiltrating immune cells

**FIGURE 9:**
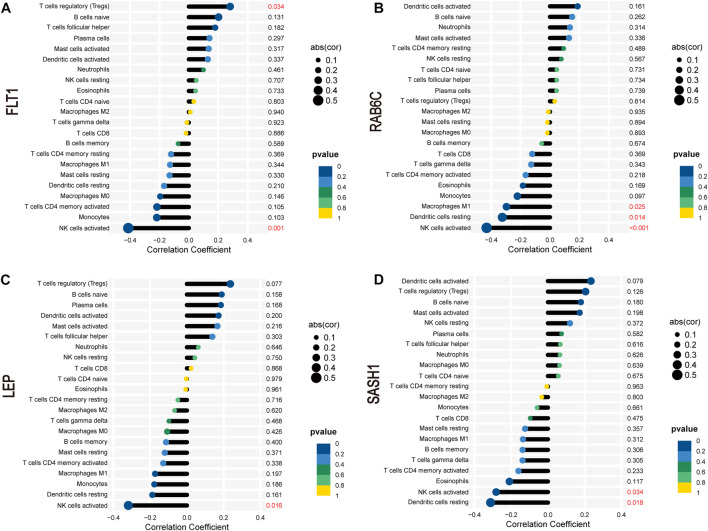
Correlation between *FLT1* **(A)**, *RAB6C* **(B)**, *LEP* **(C)**, and *SASH1* **(D)** and infiltrating immune cells in preeclampsia.

*FLT1* was positively correlated with the abundance of Tregs (r = 0.28, *p* = 0.034) but negatively correlated with that of activated NK cells (r = −0.41, *p* = 0.001; Figure 9A). *RAB6C* was negatively correlated with the abundance of M1 macrophages (r = −0.30, *p* = 0.025), resting dendritic cells (r = −0.32, *p* = 0.014), and activated NK cells (r = −0.43, *p* < 0.001; Figure 9B). *LEP* was negatively correlated with the abundance of activated NK cells (r = −0.32, *p* = 0.016; Figure 9C). *SASH1* was negatively correlated with the abundance of activated NK cells (r = −0.28, *p* = 0.034) and resting dendritic cells (r = −0.31, *p* = 0.018; Figure 9D).

## 4 Discussion

Early diagnosis of preeclampsia can improve treatment options and reduce associated morbidity and mortality (El-Dorf and Hagras, 2019). Many studies have used various biological samples, such as maternal blood, urine, or placental tissue, collected during pregnancy to determine the expression or concentration of certain diagnostic biomarkers (e.g., He et al., 2020). Further research is urgently needed to elucidate the pathophysiology of preeclampsia and determine useful diagnostic and therapeutic targets to improve diagnosis and treatment options. In the present study, we screened *LEP*, *FLT1*, *RAB6C*, and *SASH1* as potential biomarkers for preeclampsia. In addition, our results suggest that Tregs, monocytes, and activated NK cells may be involved in the development of preeclampsia. These findings can provide new insights into the pathogenesis of preeclampsia and provide new clues for obstetricians to early identify high-risk groups of preeclampsia.

In our study, placental gene enrichment in women with preeclampsia was primarily associated with the following pathways: reproductive structure development, reproduction system development, hormone transport, hormone metabolic process, and IL-17 signaling. DO analysis showed that the associated diseases were mainly those involved in the endocrine and reproductive systems and preeclampsia. Most of these outcomes are related to diseases of the reproductive system. Inflammation is the main characteristic and risk factor of preeclampsia and other hypertensive disorders complicating pregnancy. Elevated inflammatory mediators and leukocytes in peripheral blood and placental tissue can lead to abnormal uterine blood vessels and impaired placental function, especially in severe early-onset disease (Robertson et al., 2019). Consistent with our KEGG results, previous studies have reported that IL-17 is overexpressed in preeclampsia. In addition, the overexpression of IL-17 has been reported to promote the proliferation, migration, and invasion of trophoblast cells by regulating the PPAR-α/RXR-α/Wnt signaling pathway; therefore, IL-17 may be a potential therapeutic target for preeclampsia (Zhang et al., 2022). This is consistent with our KEGG results.

We identified characteristic genes of preeclampsia as *LEP, FLT1, RAB6C*, and *SASH1* using the GEO database and verified them using the GSE160888 and GSE96985 datasets. We also validated the four genes in blood samples from the GSE48424 dataset. Consistent with our findings, other studies have identified *FLT1* as a diagnostic biomarker gene (Yang et al., 2022). In the present study, *FLT1* expression was significantly increased in the placentas of patients with preeclampsia. FMS-associated tyrosine kinase 1 pseudogene 1 (*FLT1P1*) and *FLT1* have been shown to regulate trophoblast proliferation and angiogenesis in preeclampsia. Hence, the occurrence and development of preeclampsia may be owing to the abnormal regulation of *FLT1P1* and *FLT1* expression; this indicates that *FLT1P1* and *FLT1* are promising biomarkers for the diagnosis of preeclampsia (Chi et al., 2021). Leptin is involved in cell differentiation, proliferation, and immunity in various physiological states, and is mainly derived from placental and adipose tissues (Inagaki-Ohara, 2019). The upregulation of miR-18b-3p inhibits the expression of *LEP* and reduces the occurrence of preeclampsia (Huang et al., 2021). In the present study, the expression of *LEP* in the blood increased significantly in patients with preeclampsia and tended to increase in the placentas of patients with preeclampsia. Therefore, our study identified *LEP* as an important gene for preeclampsia. SASH1 is a member of the SLY family of signal adapter proteins (He et al., 2016). Previous studies using RNA sequencing have found that *SASH1* is upregulated in the placenta of patients with preeclampsia, indicating that *SASH1* plays a vital role in this organ during preeclampsia (He et al., 2016). Another study showed that *SASH1* was significantly upregulated in placentas with preeclampsia. The overexpression of *SASH1* inhibited trophoblast proliferation, migration, and invasion, but induced trophoblast apoptosis (Liu et al., 2020). In our study, *SASH1* was screened as a characteristic gene of preeclampsia, and its expression in the placenta of patients with preeclampsia was higher. However, *SASH1* showed a downward trend in the blood samples of patients with preeclampsia. Finally, *RAB6C* expression in patients with preeclampsia showed a downward trend in both placental and blood samples. However, there are no reports on the relationship between preeclampsia and *RAB6C*, which may hence be a novel molecule worthy of further study. Moreover, our results show that *LEP*, *FLT1*, *RAB6C*, and *SASH1* were significantly positively correlated, indicating a potential co-activation relationship among them or the existence of similar biological roles. The role of this correlation in preeclampsia requires further investigation.

Activated NK and resting dendritic cells showed significantly less infiltration in preeclamptic tissues than in normal tissues. *FLT1* was negatively correlated with activated NK cells. *LEP* was negatively correlated with activated NK cells. *RAB6C* was negatively correlated with resting dendritic cells, and activated NK cells. *SASH1* was negatively correlated with activated NK cells and resting dendritic cells. Decidual NK (dNK) cells are the most abundant immune cell type at the maternal-fetal interface during early pregnancy and placental formation (Cornelius and Wallace, 2019). dNK cells play a key role in spiral artery remodeling by secreting IL-8, interferon-gamma-inducible protein-10, vascular endothelial growth factor, and placental growth factor (Hanna et al., 2006). Another study found that decidual arteries have a smaller lumen diameter and damaged endothelium in NK cell-deficient mice (Greenwood et al., 2000). The most important steps in the process of placenta formation are trophoblast invasion and vascular remodeling. Decreased trophoblast cell invasion and vascular conversion resulting in poor placental perfusion may lead to the development of preeclampsia (Brosens et al., 2011). It has been demonstrated that uterine NK cell supernatant stimulates extravillous trophoblast invasion at 12–14 weeks of gestation. Increased invasiveness correlates with increased metalloproteinase-9 (MMP—9) secretion and decreased extravillous trophoblast apoptosis. MMPs are proteolytic zinc-requiring enzymes that include collagenases (Lash et al., 2010). Therefore, if the number of NK cells decreases during this period, it may lead to impaired trophoblast invasion and thus promote the development and progression of PE. dNK cells may be a useful target for the treatment of preeclampsia to ensure appropriate placental formation, vascular remodeling, and pregnancy progression (Cornelius and Wallace, 2019). These results are consistent with our findings; NK cell infiltration in the placenta of patients with preeclampsia was reduced. As for the relationship between the four genes FLT1, LEP, SASH1, and RAB6C and NK cells, no studies have been reported so far, which can be further explored in the future. Currently, there is some researches on the condition of dendritic cells in the placenta of preeclampsia. It has been suggested that the total number of dendritic cells in the placental bed of women with preeclampsia may be similar or higher (Huang et al., 2008; Hsu et al., 2012). The proportion of mature dendritic cells in the decidua of patients with preeclampsia was significantly higher than that of healthy pregnant women (Zhang et al., 2017). This is not consistent with our research. More studies are needed to explore the role of immune cell infiltration in the pathophysiological mechanism of preeclampsia.

This study has some limitations. First, some GEO datasets were not very large, and future studies should be based on larger sample sizes. Second, although four key genes have been identified as being characteristic of preeclampsia, a larger sample size is needed to validate this finding. Third, we did not identify subtypes of preeclampsia. In the future, we will further differentiate between the types of preeclampsia and perform flow sorting of the placenta to verify the type of immune cells in it so that patients can be diagnosed and managed appropriately.

In summary, *LEP*, *FLT1*, *RAB6C*, and *SASH1* were identified as potential biomarkers of preeclampsia. In addition, our findings suggest that Tregs, monocytes, and activated NK cells may participate in preeclampsia development. Thus, these immune cells are promising targets for immunotherapy in patients with preeclampsia.

## Data Availability

The original contributions presented in the study are included in the article/Supplementary Material, further inquiries can be directed to the corresponding author.
